# Laparotomy in Suppurative and Tubercular Peritonitis

**Published:** 1888-02

**Authors:** 


### *LAPAROTOMY IN SUPPURATIVE AND TUBERCULAR PERITONITIS.*

At a recent meeting of the Clinical Society of London (*The British Medical Journal,* November 5, 1887,) Mr. Barwell reported a case in which, during an acute peritonitis, following a blow upon the abdomen, he had opened the peritoneal cavity, sponged its lower portion, and washed it out with distilled water, bringing away quantities of flocculent pus. The wound was sewn close without drainage. There was rapid convalescence.

Mr. Knapp reported a case in which the abdomen was opened for a supposed ovarian cyst, and the peritoneum, mesentery, and intestines were found covered with tubercles. The cavity was washed out with warm water. The patient recovered, but had evidence of pulmonary tubercle. He alluded to the remarkable experience of Tait, who claimed uniform success so far as the operation was concerned, with complete cure in eighty per cent. of all cases of tubercular peritonitis.

Dr. Clarke reported a case of ascites with dullness over the left lung, temperature 100.2° F., pulse 120. On opening the abdomen, the intestines and peritoneum were found studded with little bodies like boiled tapioca grains. The cavity was well disinfected with a one per cent. carbolic solution. Convalescence was uninterrupted, and in six weeks the patient seemed perfectly well.

Mr. Treves thought most surgeons would prefer to use a drainage tube after these operations. He referred to a record recently published in Germany, of ninety-seven cases of draining the abdomen for peritonitis, and to Kussmaul's paper comprising thirty cases, with six others subsequently added, making thirty-six cases with six deaths, two from the operation, and four from general tuberculosis. A discussion as to methods and indications for the operation followed.

Professor Breisky reports (*Centralblatt für gesammte Therapie* June, 1887,) a case of operation for supposed ovarian tumor, in which widespread intestinal tuberculosis was found, as was demonstrated by examination of a small section of the peritoneum. The wound healed by first intention, and the patient improved in general health, although a pulmonary tuberculosis, which had existed prior to the operation, was unaffected by it.

Dr. E. Ceppi reported (*Rev. med. de la Suisse Romande*, May 16, 1887,) a case of chronic purulent peritonitis, cured by abdominal incision with drainage. The so-called Neisser's gonococci were found in the peritoneal exudation.

Besnier reported, at a recent meeting of the Paris Society of Medicine, a case of suppurative puerperal peritonitis, in which the abscess was encysted. Aspiration, the evacuation of five and a-half quarts of pus, iodine and collodion externally, and the free use of tonics, resulted in a cure.

